# Supplementary material for: DAAM mediates the assembly of long-lived, treadmilling stress fibers in collectively migrating epithelial cells in Drosophila
Source: eLife. 2021 Nov 23;10:e72881. doi: 10.7554/eLife.72881 (PMC8610420; doi:10.7554/eLife.72881)
Supplement: Supplementary file 1. — Detailed list of the genotype corresponding to each figure panel, also showing the temperature at which females were matured on yeasted food prior to dissection. [file elife-72881-supp1.docx]

**Supplementary File 1. Experimental genotypes**

| Figure | Panel | Genotype | °C |
| --- | --- | --- | --- |
| **1** | A-B | *w^1118^* | 25 |
| **2** | A-C, F-I | *sqh^Ax3^/w;; sqh-GFP* | 25 |
|  | D-E | *sqh^Ax3^/w; UAS-Pax-GFP/+; da****-****Gal4/sqh-mCh* | 25 |
| **2 – S1** | A | w/*y w hsFLP/+;; UAS-Ftractin-Tom/act5c>>Gal4* | 25 |
|  | B | *y w/y w hsFLP;; UAS-Lifeact-GFP/+; act5c>>Gal4/+* | 25 |
|  | C | *w/y w hsFLP;; UAS-Moe-ABD-mCh/ act5c>>Gal4* | 25 |
|  | D | *w/y w hsFLP; UAS-Utr-ABD-GFP/+; act5c>>Gal4/+* | 25 |
| **2 – S2** | A-B | *sqh^Ax3^/w;; sqh-GFP* | 25 |
| **3** | A | *w/y* w*; tj-Gal4/UAS-Pax-GFP* | 25 |
|  | B, E-G | *w; Pax-GFP* | 25 |
|  | C | *w;;Talin-GFP* | 25 |
|  | D | *w mys-GFP* | 25 |
| **3 – S1** | - | *w; Pax-GFP* | 25 |
| **4** | A-E | *sqh^Ax3^/w; UAS-Pax-GFP/+; da-Gal4/sqh-mCh* | 25 |
| **5** | A left | *w; Pax-GFP/+; sqh-mCh/+* | 25 |
|  | A middle, B | *w; Pax-GFP/+; sqh-mCh/+* | 25 |
|  | A right, C | *w/y w; tj-Gal4/Pax-GFP; sqh-mCh/UAS-Abi RNAi^NIG: 9749R-3^* | 25 |
|  | D | *y sc* v sev/y w hsFLP; UAS-Pax-GFP/ +; UAS-Sra1 RNAi^TRiP.HMS01754^/act5c>>Gal4* | 25 |
|  | E | *y sc* v sev/y w hsFLP; act5c>>Gal4, UAS-RFP/+; UAS-Sra1 RNAi^TRiP.HMS01754^/sqh-mCh* | 25 |
| **6** | A, D | *w/y w hsFLP;; UAS-Abi RNAi^NIG: 9749R-3^/act5c>>Gal4, UAS-RFP* | 25 |
|  | B, D | *w/y w hsFLP; UAS-DAAM RNAi^KK102786^/+; act5c>>Gal4, UAS-RFP/+* | 25 |
|  | C, D | *w/y w hsFLP;; UAS-pTWFlag-C-DAAM/+; act5c>>Gal4, UAS-RFP/+* | 29 (2d) |
|  | D | *hsFLP Ubi-mRFP-nls FRT19A/DAAM^A^ FRT19A* | 25 |
|  | D | *hsFLP Ubi-mRFP-nls FRT19A/DAAM^Ex68^ FRT19A* | 25 |
|  | E | *hsFLP* *Ubi-mRFP-nls FRT19A/DAAM^A^ FRT19A* | 25 |
| **6 – S1** | A, C | *y sc* v sev/y w hsFLP;; UAS-Sra1 RNAi^TRiP.HMS01754^/act5c>>Gal4, UAS-RFP* | 25 |
|  | B, C | *w/y w hsFLP; UAS-Ena RNAi^GD8910^/+; act5c>>Gal4, UAS-RFP/+* | 25 |
|  | D | *w/y w hsFLP; UAS-RNAi /+; act5c>>Gal4, UAS-RFP/+*  *or w/y w hsFLP;; UAS-RNAi/act5c>>Gal4, UAS-RFP*  *(exact RNAi lines used are noted in figure)* | 29 (2d) |
|  | E | *Ubi-mRFP-nls FRT19A/DAAM^A^ FRT19A; tj-Gal4, UAS-Flp/ UAS-RNAi or*  *Ubi-mRFP-nls FRT19A/DAAM^A^ FRT19A; tj-Gal4, UAS-Flp/+; UAS-RNAi/+*  *(exact RNAi lines used are noted in figure)* | 25 |
| **7** | A, B | *DAAM-GFP/w^1118^* | 25 |
|  | C | *DAAM^Ex68^ FRT19A/hsFLP Ubi-mRFP-nls FRT19A* | 25 |
|  | D | *DAAM^A^ FRT19A/hsFLP Ubi-mRFP-nls FRT19A; tj-Gal4/+* | 25 |
|  | E control | *w/y w; Cyo/UAS-DAAM RNAi^KK102786^; sqh-mCh/+* | 25 |
|  | E experimental | *w/y w; tj-Gal4/UAS-DAAM-DAAM RNAi^KK102786^; sqh-mCh/+* | 25 |
|  | F depletion | *w/y w hsFLP; UAS-DAAM RNAi^KK102786^; TalinGFP/act5c>>Gal4, UAS-RFP* | 29 (2d) |
|  | F overexpression | *w/y w hsFLP; UAS-pTWFlag-C-DAAM/+; TalinGFP/act5c>>Gal4, UAS-RFP* | 25 |
|  | G control | *w/y w; tj-Gal4/+; UAS-Dcr2/+* | 25 |
|  | G experimental | *w/y w; tj-Gal4; UAS-Dcr2/UAS-DAAM RNAi^KK102786^* | 25 |
| **8** | A | *DAAM-GFP/w^1118^* | 25 |
|  | B | *DAAM^A^ FRT19A/hsFLP Ubi-mRFP-nls FRT19A*  *and DAAM^Ex68^ FRT19A/hsFLP Ubi-mRFP-nls FRT19A* | 25 |
|  | C | *w/y w hsFLP; +; UAS-Dia RNAi^GD9442^/act5c>>Gal4, UAS-nls-RFP* | 25 |
|  | D | *(same as the four genotypes listed in 8E below)* | 29 (2d) |
|  | E left | *w^1118^; Cy2-Gal4/+* | 29 (2d) |
|  | E left middle | *w^1118^; Cy2-Gal4/UAS-DAAM RNAi^KK102786^* | 29 (2d) |
|  | E right middle | *w^1118^; Cy2-Gal4/+; UAS-Dia RNAi^KK101745^* | 29 (2d) |
|  | E right | *w^1118^; Cy2-Gal4/+; UAS-Dia RNAi^GD9442^* | 29 (2d) |
| **Videos** | 1, 2 | *sqh^Ax3^/w;; sqh-GFP* | 25 |
|  | 3, 4 | *sqh^Ax3^/w; UAS-Pax-GFP/+; da-Gal4/sqh-mCh* | 25 |
|  | 5 | *w; Pax-GFP/+; sqh-mCh/+* | 25 |
